# Online Social Networks for Crowdsourced Multimedia-Involved Behavioral Testing: An Empirical Study

**DOI:** 10.3389/fpsyg.2015.01991

**Published:** 2016-01-11

**Authors:** Jun-Ho Choi, Jong-Seok Lee

**Affiliations:** School of Integrated Technology and Yonsei Institute of Convergence Technology, Yonsei UniversityIncheon, South Korea

**Keywords:** crowdsourcing experiment, social network, behavioral testing, music information retrieval, social media advertising, voluntary involvement

## Abstract

Online social networks have emerged as effective crowdsourcing media to recruit participants in recent days. However, issues regarding how to effectively exploit them have not been adequately addressed yet. In this paper, we investigate the reliability and effectiveness of multimedia-involved behavioral testing via social network-based crowdsourcing, especially focused on Facebook as a medium to recruit participants. We conduct a crowdsourcing-based experiment for a music recommendation problem. It is shown that different advertisement methods yield different degrees of efficiency and there exist significant differences in behavioral patterns across different genders and different age groups. In addition, we perform a comparison of our experiment with other multimedia-involved crowdsourcing experiments built on Amazon Mechanical Turk (MTurk), which suggests that crowdsourcing-based experiments using social networks for recruitment can achieve comparable efficiency. Based on the analysis results, advantages and disadvantages of social network-based crowdsourcing and suggestions for successful experiments are also discussed. We conclude that social networks have the potential to support multimedia-involved behavioral tests to gather in-depth data even for long-term periods.

## Introduction

Nowadays, online crowdsourcing is one of the widely used methods for conducting behavioral experiments with large numbers of people. In comparison to the traditional way of performing oﬄine experiments, crowdsourcing on the Internet enables researchers to collect massive amounts of data and requires less effort to recruit participants, set up the experimental environments, and run the experiments (Wu et al., 2013). In addition, it has been shown that the quality of experimental results from crowdsourcing is almost the same as that from oﬄine-recruited participants (Casler et al., 2013).

One of the important issues in conducting crowdsourcing-based experiments is ensuring the quality of experimental data. It is crucial to determine a proper way to motivate people to participate in the experiment, which includes financial reward, altruism, enjoyment, reputation, and implicit work (Quinn and Bederson, 2011). For example, Amazon Mechanical Turk (MTurk) is a financial reward-based crowdsourcing marketplace in which one can post small tasks, called Human Intelligence Tasks (HITs), and people who fulfill those tasks get monetary reward from the task provider. reCAPTCHA is an example of implicit crowdsourcing work in which users are asked to type both scanned and distorted words in order to prevent automated programs that perform abnormal activities such as registering numerous user accounts (Von Ahn et al., 2008). Enjoyment-based applications inspired by gaming also exist in many fields, such as music annotation (Barrington et al., 2012), image labeling (Von Ahn and Dabbish, 2004), and protein structure prediction (Cooper et al., 2010).

Many crowdsourcing-based research studies have relied on financial reward to motivate participants due to its simplicity and similarity to traditional oﬄine experiments. MTurk – which offers an efficient interface to induce mass participation and give financial reward directly to participants, called workers, after completion of the experiment’s tasks – has been widely used. It has been shown that MTurk-based experiments have not only almost the same reliability of results as traditional oﬄine experiments but also higher immediacy for finishing given tasks (Buhrmester et al., 2011; Holden et al., 2013). In addition, the characteristics and efficiency of financial reward-based experiments have been analyzed extensively in the literature. For example, Buhrmester et al. (2011) evaluated the efficiency of MTurk in terms of quality of experimental results with respect to the amount of payment. Paolacci and Chandler (2014) showed that enlightening workers is more important to obtaining high-quality results than raising payment rates.

As alternative crowdsourcing media, online social networks have often been considered in recent days. Facebook, one of the most popular social network services with more than 1 billion daily active users around the world (Facebook, 2015), has been used in several experiments, such as those testing quality of experience (QoE) for videos (Gardlo et al., 2012) and photo album summarization (Vajda et al., 2011). Facebook allows researchers to retrieve users’ personal information and interests, so it is easy to recruit participants who have specific interests or to analyze experimental results with respect to demographic characteristics. Moreover, it is not required to pay participants directly, so motivations other than monetary reward can be chosen to prevent issues arising from the discrepancy between the incentives and experimental purpose. Despite these advantages, research studies analyzing the characteristics and efficiency of social networks, which are pivotal for the successful design of crowdsourcing experiments, are rarely found. As with crowdsourcing experiments based on financial reward, it is necessary to analyze the properties of social network-based crowdsourcing to help researchers plan their own experiments tactically to maximize both the efficiency and quality of data.

Recently, we conducted a crowdsourcing-based experiment called “EvoTunes” (Choi and Lee, 2014). It aimed at collecting data regarding user satisfaction with given music playlists that could be used for automatic playlist recommendations. Each participant listened to two specific songs in a row whose order was determined by a recommendation algorithm and provided satisfaction ratings. This procedure was repeated with different song pairs for many participants. When a sufficient amount of feedback data was gathered, the recommendation algorithm calculated the probability of satisfaction for each song-to-song transition to generate new playlists expected to have higher levels of satisfaction.

Optimization of the recommendation process required a large amount of experimental data on users’ satisfaction with song transitions. Therefore, it was important to design an efficient and reliable way of conducting a crowdsourcing-based experiment. For this, MTurk was the first candidate as the most popular crowdsourcing medium. However, two problems arose: first, the gathered data could have been largely compromised due to unfaithful responses or cheating if workers pursued only monetary reward, and it was extremely difficult to detect such responses due to the lack of ground-truth data. Second, the environment of music listening through MTurk was significantly different from real music listening situations; thus, even if the gathered data were reliable, they might not have been suitable for playlist generation.

**FIGURE 1:**
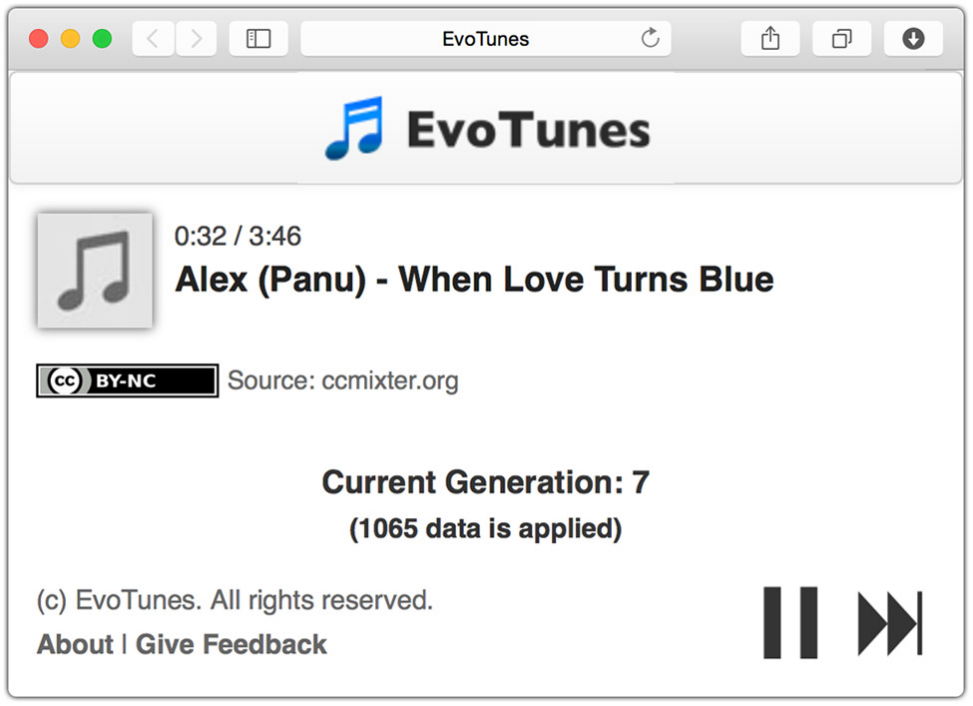
**Screenshot of music player in our web-based experiment interface**.

To address these issues, we decided to create our own web-based experiment interface. Participants listened to music clips suggested by the recommendation algorithm through the simple music player shown in **Figure 1**, which is similar to that of common music streaming services. If two particular songs were played until the end, the listener was considered satisfied with the playlist; however, if the listener pressed the “skip” button to listen to another song, the listener was considered dissatisfied. This interface was able to provide a natural music listening experience that minimized the feeling of being involved in an experiment.

Since the experiment required voluntary participation, we needed an appropriate way to recruit participants interested in music streaming services. In the end, we decided to exploit an online social network, Facebook, which provides a fully customizable means of public relations, including advertisement and promotional pages^1^. We recruited a sufficient number of participants over 30 days through Facebook and ran our experiment successfully. Moreover, we collected a massive amount of data regarding users’ behaviors on both Facebook and the experiment interface.

Based on the collected data, in this paper, we investigate the efficiency and usability of online social networks to attract participants for crowdsourcing experiments, especially focusing on the following research questions.

• How can participants be effectively attracted for crowdsourcing experiments in online social networks?

Since monetary reward are not given to participants, ways of attracting participants is a critical issue in social network-based crowdsourcing experiments. For this, advertisement, which is a unique tool in social networks such as Facebook, can be exploited. Previous studies including Balfe et al. (2012) concluded that it is hard to attract participants via advertisement of online social networks due to low response rates. However, they may be still exploitable if a sufficiently large number of participants can be hired at a reasonable cost. Moreover, different types of advertisement have not been compared in existing studies. In this paper, different tools for advertisement on Facebook are tested and compared in terms of effects and efficiency.

• How can particular groups of users be appropriately targeted in online social networks?

Users’ behaviors within a social network-based crowdsourcing experiment may vary with the users’ demographic characteristics, such as gender and age group. There have been studies examining gender-dependence of participation in crowdsourcing (e.g., Thornton et al., 2015), but other characteristics such as age have been rarely studied. Understanding such variability is helpful for designing a recruiting strategy to effectively target particular groups of users suited for the goal of the experiment. We try to answer this question by examining the demographic characteristics of the participants in our Facebook-based experiment.

• Do online social networks offer reliability and efficiency comparable to other recruitment methods?

To take advantage of online social networks to attract participants for reliable crowdsourcing experiments, their efficiency must first be proved. As aforementioned, this has not been studied well in literature. We analyze how efficient it is to use online social networks for multimedia-involved crowdsourcing experiments in comparison to other media such as MTurk.

The methods used to conduct our experiment are described in Section “Materials and Methods.” The analysis of the experimental results in various aspects is shown in “Results.” In “Discussion” discusses the outcomes, compares our approach with existing crowdsourcing methods, discusses the advantages and disadvantages of online social networks, makes suggestions for successful recruitment using social networks, and finally presents our conclusions.

## Materials and Methods

### Procedure

**FIGURE 2:**
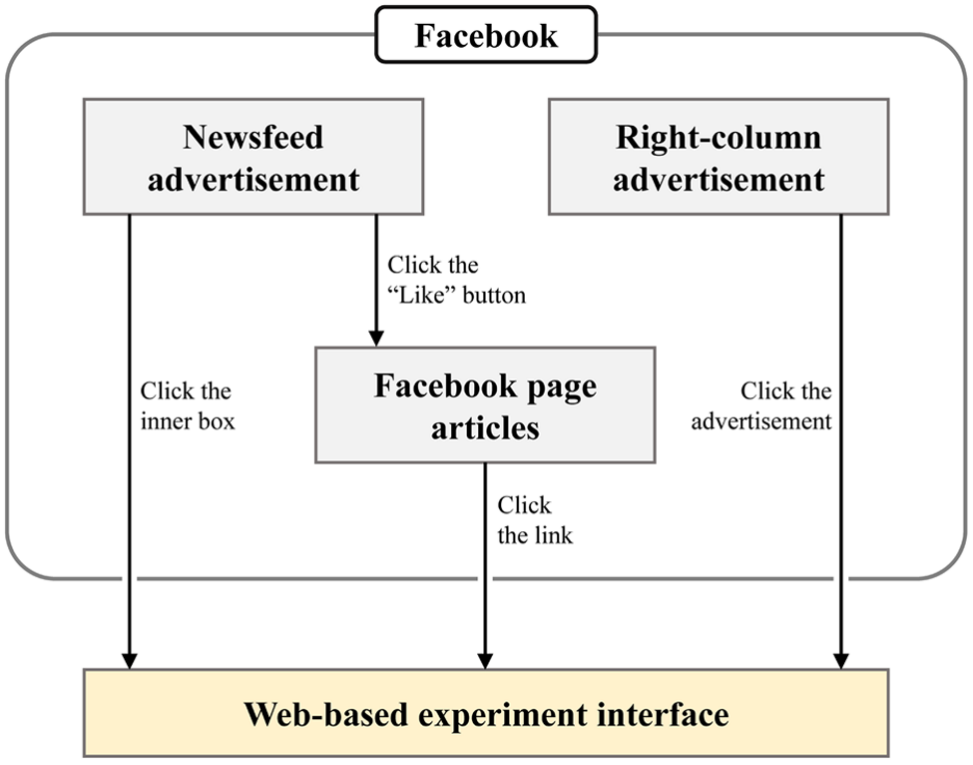
**Overview of routes directing participants to experiment interface using Facebook**. Three attractions (marked as gray boxes) are used for attracting users to the web-based experiment interface via actions described on the right side of the arrows.

**Figure 2** shows an overview of how participants were attracted in our experiment. The two components that we exploited were advertisements of our experiment interface using the Facebook advertisement system and articles posted on the Facebook page for the interface. The advertisements guided users to visit our experiment interface directly or to click the “Like” button to subscribe to our Facebook page. For those who subscribed to the page, new articles on the page were shown on their main homepage that encouraged them to participate in the experiment repeatedly. The study was carried out in compliance with the ethical recommendations in the Declaration of Helsinki, and informed consent was provided on the experiment interface. In addition, the advertisement data were aggregated on an anonymous basis to abide by the Facebook advertising policies.

#### Facebook Advertisement

The Facebook advertisement system allows advertisers to choose various options, such as targeting users who have specific interests and locating an appropriate position to display the advertisement. Once the options are configured, Facebook randomly selects users within the targeted user group to show advertisements. To maximize the efficiency of our advertisement, we targeted users who were over the age of 18, had expressed interest in music-related topics, and lived in English-speaking countries including the United States, United Kingdom, Canada, Australia, and the Philippines.

Advertisers need to set a daily budget and choose one of the two charging options: charge per 1,000 exposures or charge per click. Because inducing users to click the advertisement was more important than exposing the advertisement in our case, we chose the click-based charging option. In this option, the maximum cost per click (CPC) of the advertisement can be set. Then, Facebook automatically decides an optimized CPC based on the maximum costs of other advertisements having similar targets. During our experiment, we often altered the daily budget of our advertisement so that the number of exposures changed accordingly. Here, we set only the total budget for both advertisement types; the budget for each advertisement type was not specified.

**FIGURE 3:**
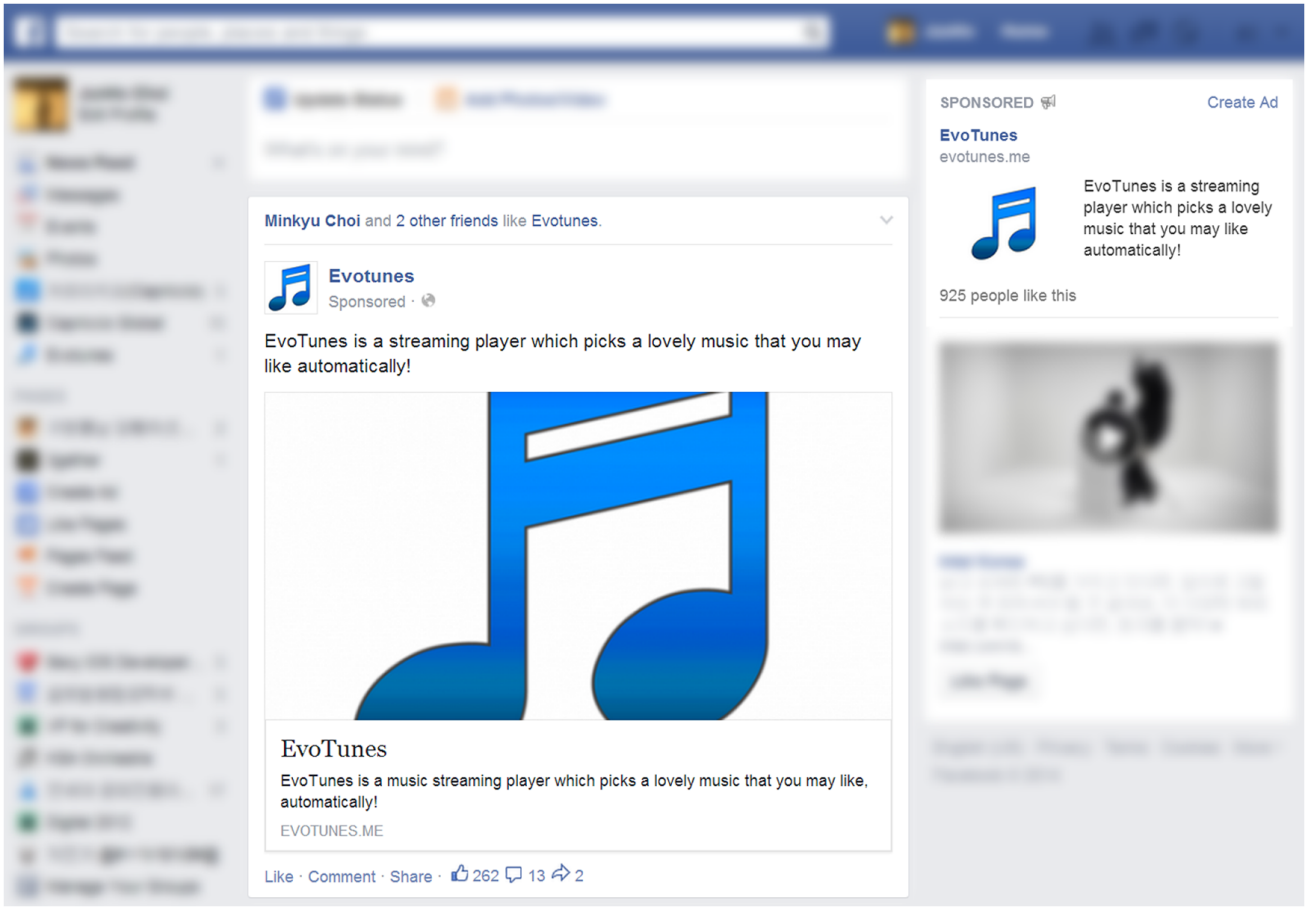
**Two types of Facebook advertisements: newsfeed advertisement on main homepage of Facebook and right-column advertisement on right side of Facebook site**.

Although Facebook provides detailed targeting options, such as gender, education level, and so on, we did not set any other particular targeting configurations except for those mentioned above in order to prevent any bias in the experimental results and enable general conclusions to be drawn from the results. For example, we set the age range as over 18, which is the default targeting option. In addition, we used both types of Facebook advertisements appearing in the desktop version of Facebook, as shown in **Figure 3**.

##### Newsfeed advertisement

A newsfeed advertisement appears directly on a user’s main homepage of Facebook, called “News Feed.” It is marked as “Sponsored” to be distinguished from posts published by friends. Users could click the inner box of the advertisement containing a thumbnail image, title, description, and link to go to our experiment interface or click the “Like” button at the bottom to subscribe to our Facebook page. The number of “Likes” for our Facebook page was shown next to the “Like” button. We activated this type of advertisement for 30 days with adjustment of the daily cost, *N = 30, M = $7.45, SD = $8.45*.

##### Right-column advertisement

A right-column advertisement appears in the “Sponsored” section on the right side of the Facebook site. Unlike the newsfeed advertisement, this type of advertisement did not contain the “Like” button for our Facebook page. Users could click any part of the advertisement to launch our experiment interface. We activated this type of advertisement only for 7 days due to its inefficiency (see Differences by Advertisement Type) with adjustment of the daily cost, *N = 7, M = $5.18, SD = $5.73*.

#### Facebook Page

Facebook allows users to create pages for promoting various goods and services (e.g., a website, entertainment program, shoe brand). Any Facebook user can create a page, and there are no fees. Posts can be written on such a page, which are shown on the “News Feed” of the users who have pressed the “Like” button of the page.

We created a page on the day we launched our experiment interface and maintained it for more than 30 days. Each article that we regularly posted on the page contained the link to the interface to encourage repeated participation in our experiment. The typical content of the articles was as follows: “The algorithm for music recommendation has been updated. [the link to the interface].”

### Measures

#### Statistics from Facebook

We obtained the following statistics from the daily reports produced by Facebook Ads Manager, grouped by advertisement type, age, and gender:

• Reaches: number of users our advertisements reached
• Exposures: total number of times our advertisements were exposed
• Clicks: number of clicks of link to the experiment interface on advertisements
• Cost per mille (CPM): average cost per 1,000 exposures of advertisements
• Click-through rate (CTR): number of clicks of link per exposure
• Cost per click: average CPC of link on advertisements

In addition, we determined the number of people who pressed the “Like” button on our Facebook page from the daily reports of users’ activities offered by Facebook.

#### User Statistics of Experiment Interface

Our experiment interface was designed in such a way that participants could login to the interface with their Facebook accounts in order to eliminate the inconvenience of creating new accounts. This enabled us to retrieve personal information about each user. We collected only basic personal information because retrieving additional information requires the users’ permission, which might have been unacceptable for some users and consequently lowered the number of participants (Vajda et al., 2011).

Every activity in our experiment interface was tracked in terms of type and time stamp, including logging in and pressing the music player buttons (e.g., play, pause, skip).

## Results

During the experimental period, our Facebook advertisement cost $266.27 in total. It had 1,122,751 reaches, 4,343,736 exposures, and 2,431 clicks, which resulted in a CPM of $0.06, CTR of 0.056%, and CPC of $0.11. At the same time, our Facebook page received 768 “Likes.” A total of 395 participants joined our experiment interface, the total number of their activities was 3,145, and the average time spent on the experiment interface was 18.59 min.

### Daily Flows of Participants

**FIGURE 4:**
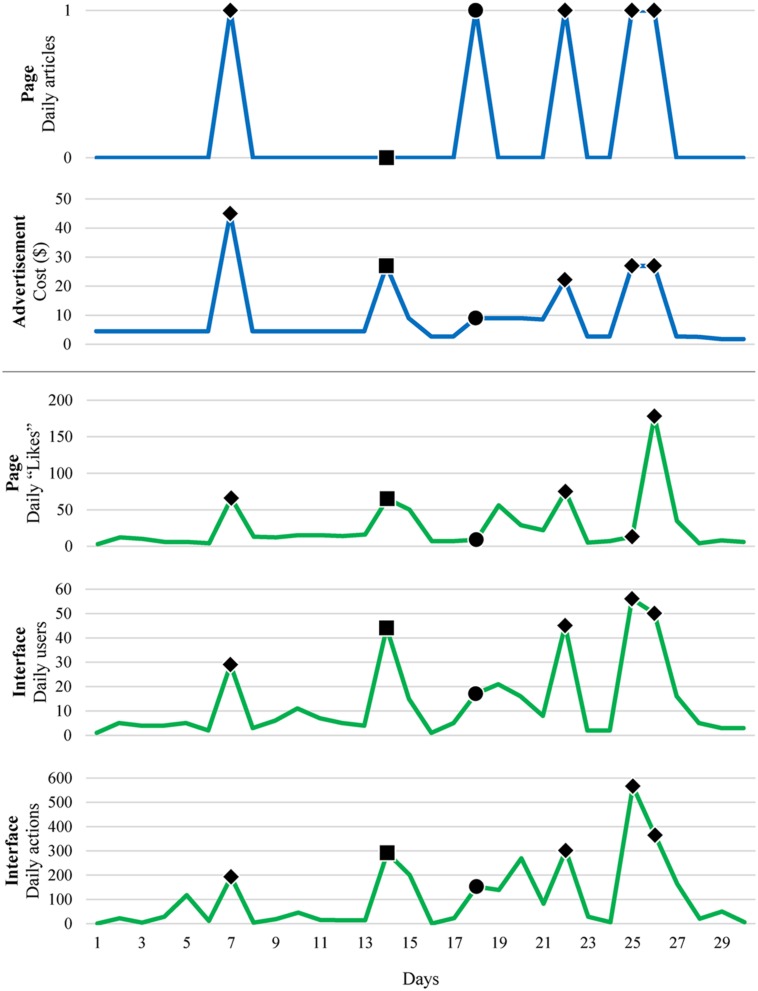
**Daily statistics of participants’ actions in relation to changes in promotional factors.** The days when articles were posted are marked with circles (●), and the days when the advertisement cost was set high are marked with squares (■). The days when both articles were posted and the cost was set high are marked with diamonds (♦).

**Figure 4** shows the daily statistics of promotional elements and participants’ actions. The number of articles posted on our Facebook page and the cost of the Facebook advertisement are shown at the top in blue. The number of “Likes” that the Facebook page received, the number of users who registered on our experiment interface, and the number of actions of the participants are shown at the bottom in green. The days when articles were posted are marked with circles (●), and the days when the advertisement cost was set high are marked with squares (■). The days when both articles were posted and the cost was set high are marked with diamonds (♦).

It is noticeable that the number of participants’ actions was significantly higher when we promoted our experiment. For example, when we posted an article on our Facebook page on Days 7, 18, 22, 25, and 26 and when we set the daily budget of the advertisement high on Days 7, 14, 22, 25, and 26, the number of actions prominently increased. When Day 18 (an article was posted on the Facebook page without budget adjustment) and Day 14 (the daily budget was set high without posting an article) were compared, the latter was found to be more effective in facilitating users’ actions, which directly influenced the number of exposures.

**Tables 1** and **2** present statistical analysis results regarding the relationship between attractions and responses. As shown in the tables, both articles posting and increasing the advertisement cost have a strong influence on every type of response, especially the number of daily users in our experiment interface.

**Table 1:** *z*-statistics and *p*-values of one-sided Wilcoxon rank-sum tests comparing responses (number of daily “Likes” of Facebook page, number of daily users of experiment interface, and number of daily actions in interface) when articles were posted on Facebook page (*N = 5*) and those when no articles were posted (*N = 25*).

Page: Daily “Likes”	Interface: Daily users	Interface: Daily actions
-2.090 (*p* = 0.018)	-3.296 (*p* < 0.001)	-3.062 (*p* = 0.001)

**Table 2:** Correlation coefficients (Kendall’s τ) and *p*-values between advertisement cost and responses (number of daily “Likes” of Facebook page, number of daily users of experiment interface, and number of daily actions in interface).

Page: Daily “Likes”	Interface: Daily users	Interface: Daily actions
0.558 (*p* < 0.001)	0.637 (*p* < 0.001)	0.511 (*p* < 0.001)

### Differences by Advertisement Type

**FIGURE 5:**
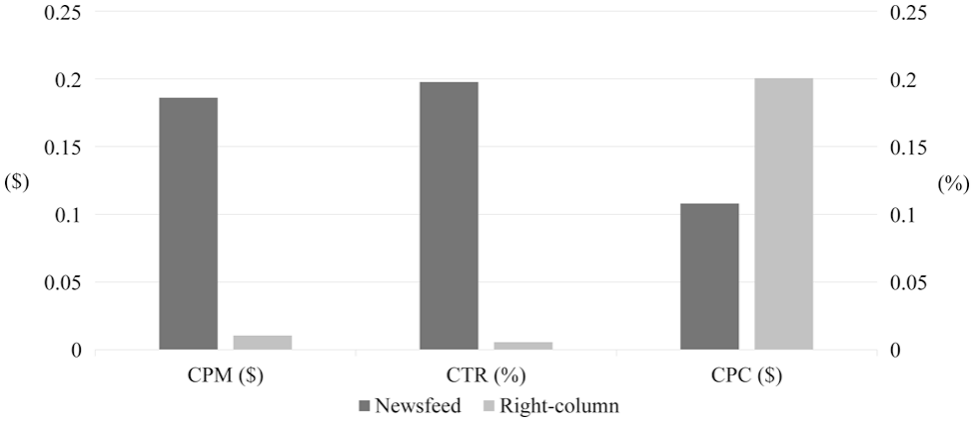
**Comparison of newsfeed advertisement and right-column advertisement in terms of CPM, CTR, and CPC**.

For the two types of Facebook advertisements, CPM, CTR, and CPC are compared in **Figure 5**. As shown in the figure, the CPM of the right-column advertisement is much smaller than that of the newsfeed advertisement; the right-column advertisement was exposed to users about 19 times more often than the newsfeed advertisement for the same cost. However, the CTR of the right-column advertisement is much smaller than that of the newsfeed advertisement.

As a result, the CPC of the newsfeed advertisement (*N = 30, M = 0.11, SD = 0.03*) is lower than that of the right-column advertisement (*N = 7, M = 0.21, SD = 0.04*). A one-sided Wilcoxon rank-sum test also confirms the statistical significance of this difference (*z = -3.897, p < 0.001*). It is because the CTR of the newsfeed advertisement is significantly higher than that of the right-column advertisement (*0.198% > 0.006%*) even though the latter has a lower CPM (*$0.19 > $0.01*). This implies that the newsfeed advertisement is more effective for attracting participants, while the right-column advertisement is better if frequent exposures of certain information are the aim. Because we needed to maximize the number of clicks rather than the number of exposures, we stopped delivering the right-column advertisement after 7 days and focused our budget on the newsfeed advertisement thereafter.

### Differences by Gender

In total, the newsfeed advertisement was exposed 603,660 times to 302,112 males and 474,819 times to 254,393 females. From these statistics, we examine gender differences in terms of daily CPM, CTR, and CPC for the newsfeed advertisement via one-sided Wilcoxon signed-rank tests. The test result for CTR (*z = -2.016, p = 0.022*) shows that women clicked more than men for the same number of exposures to the advertisement. In addition, the results for both CPM (*z = 3.949, p < 0.001*) and CPC (*z = 2.715, p = 0.003*) strongly suggest that a higher budget was required to attract men than women.

**FIGURE 6:**
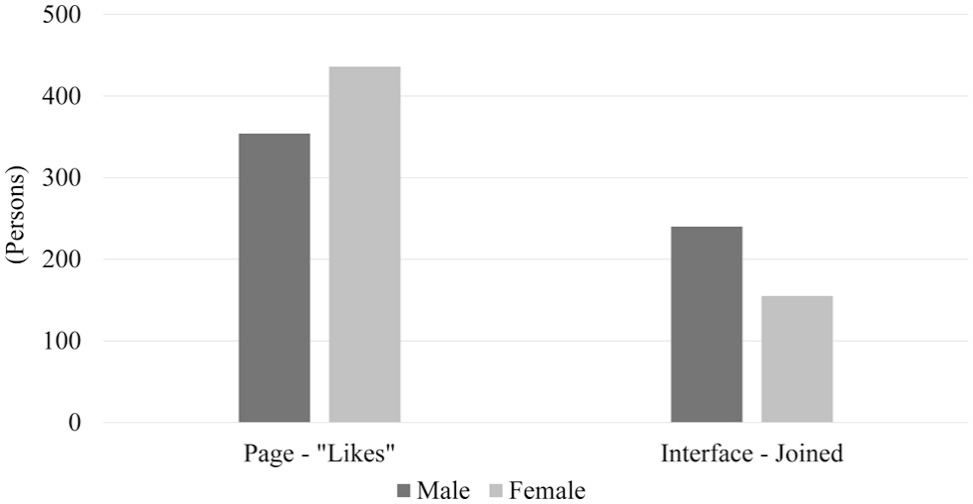
**Gender differences in two responses: number of page “Likes” and number of people who joined our experiment interface**.

**Figure 6** shows how differently males and females acted in response to the Facebook advertisements. It is observed that females clicked the “Like” button of the Facebook page 23.2% more than males, while males actually joined the experiment interface 54.8% more than females. One-sided Wilcoxon signed-rank tests also confirm the statistical significance of the gender differences for both the number of page “Likes” (*z = -2.400, p = 0.008*) and the number of participants who joined the interface (*z = 3.491, p < 0.001*). These imply that females were more active on Facebook than males. However, males showed a higher tendency to use the service immediately, which required going outside of Facebook.

### Differences by Age

**FIGURE 7:**
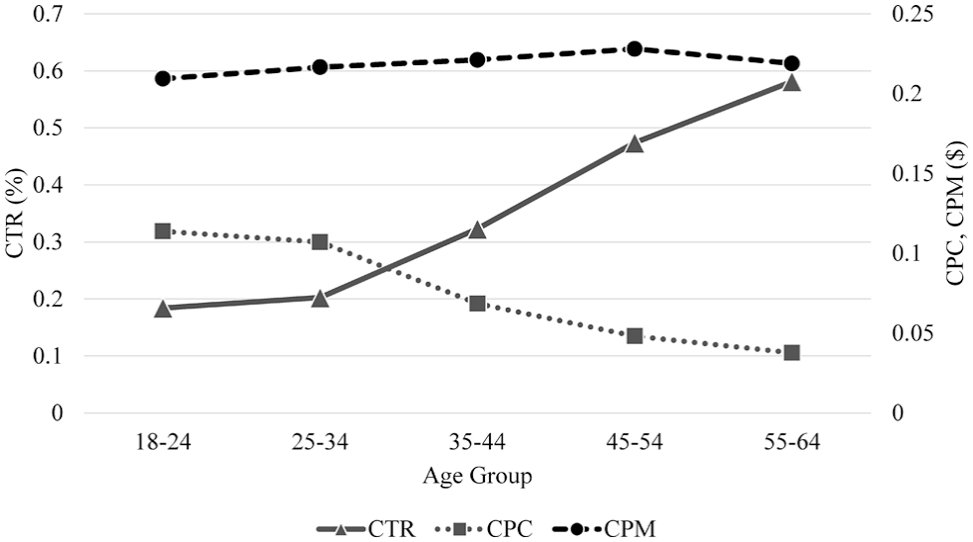
**Comparison of age groups in terms of CTR, CPC, and CPM for newsfeed advertisement**.

**Figure 7** compares different age groups (as defined by Facebook) for the newsfeed advertisement in terms of CTR, CPC, and CPM. It can be seen that CTR increases and CPC decreases with increasing age. Kruskal–Wallis tests also confirm significant differences between the age groups for CTR (*χ = 22.052, df = 4, p < 0.001*) and CPC (*χ = 20.433, df = 4, p < 0.001*) but not for CPM (*χ = 1.878, df = 4, p = 0.758*).

## Discussion

Previous studies have raised concerns about the inefficiency of using online social networks to attract participants. Balfe et al. (2012) noted that it is difficult to recruit participants from social networks, because they have very low response rates. For example, Levine et al. (2011) got a response rate of 0.024% from MySpace, and Lord et al. (2011) and Kapp et al. (2013) reported that the CTR of their Facebook advertisement ranged from 0.02 to 0.04% and 0.024 to 0.033%, respectively. In our case, we obtained a CTR of 0.056% on average, which is slightly higher than those in the previous studies but has the same order of magnitude. However, we argue that it is more meaningful to consider the cost than the response rate in evaluating the efficiency of online social networks for crowdsourcing. When a crowdsourcing problem is dealt with, a certain minimum number of participants or responses is usually required in order to be able to conduct reliable statistical analyses. For a fixed value of CTR, this can be achieved via increasing exposures of the advertisement in social networks by, for example, increasing the budget for the advertisement. Of course, the user pool the advertisement will reach must be sufficiently large, but this will be mostly satisfied due to the extremely large number of Facebook users. In our experiment, the overall CPC was $0.11. In Section “Comparison with other Crowdsourcing Projects,” we will show that this result corresponds to reasonable cost efficiency that is comparable to other crowdsourcing experiments using MTurk.

We showed that both advertisements and page articles were able to attract participants, and the former was more effective. When the newsfeed type and right-column type of advertisements were compared, our results showed that the former was more effective than the latter, recording CPMs of 0.198 and 0.006%, respectively. Thornton et al. (2015) recruited participants for a self-report questionnaire about drugs and alcohol via the right-column advertisement of Facebook. They achieved a CTR of 0.054%, which is significantly higher than that of our experiment, because they advertised the chance to win a prize as a reward for participation. Note that, however, such a monetary reward-based approach was not applicable in our case due to the reasons mentioned in the introduction. Instead, we raised the CTR to 0.056% on average by employing the newsfeed advertisement. In the study of Lord et al. (2011), another method of improving the efficiency of the Facebook advertisement was shown. They did not offer any incentive to participants but achieved a CTR of up to 0.04% by targeting a specific group of interest for the Facebook advertisement (i.e., the advertisement was shown only on the Facebook pages of 24 selected colleges and universities).

We also note that the users’ behavioral patterns with respect to their demographic characteristics in our experiment are in line with existing findings, particularly for gender differences. It is known that females usually engage in more Facebook activities than males (Hampton et al., 2012), but males participate in experiments more than females (Thornton et al., 2015). Our experimental results also follow the same patterns: women engaged in more Facebook activities but less external engagements. Regarding age differences, our results show a higher CTR as age increases. This seems to be because older people either have higher interest in the advertisement or have more difficulty in distinguishing advertisements from posts published by their friends. It is hard to compare this result with existing findings due to the lack of comparable studies. McAndrew and Jeong (2012) reported less Facebook activities of older people in terms of, for example, number of hours on Facebook, number of friends, and photo activities, but such activities are very different from clicking advertisements.

### Comparison with other Crowdsourcing Projects

To examine the suitability of attracting participants via online social networks, we compare our analysis results with existing crowdsourcing projects, especially those that recruit participants via MTurk and whose topics are related to music information retrieval. **Table 3** shows the comparison of our study and three other studies in terms of experiment type, crowdsourcing medium, data quality-control method, number of participants, number of accepted HITs, number of rejected HITs, average cost per accepted HIT, and completion time per HIT.

**Table 3:** Comparison of our experiment with existing MTurk-based crowdsourcing experiments.

	EvoTunes	Lee (2010)	Mandel et al. (2010)	Speck et al. (2011)
Topic	Music recommendation	Music similarity judgment	Music tagging	Musical mood annotation
Medium	Facebook	MTurk	MTurk	MTurk
Quality control	Statistical filtering	Duplicated questions	Statistical filtering	Statistical filtering
Number of participants	395	N/A	209	272
Accepted HITs	935^∗^	583	2566	634
Rejected HITs	2210^∗^	464	305	756
Cost per accepted HIT ($)	0.28^∗^	0.22	≥0.03	0.54
Completion time per HIT (s)	248.2^∗^	N/A	N/A	≥330

*^∗^Human Intelligence Tasks are defined only in MTurk, and in our case, HITs are not clearly defined. However, for comparison, we consider each action in our experiment (e.g., listening to as long until the end, pressing the skip button) as a HIT, based on which we estimate HIT-related statistics.*

Lee (2010) examined the efficiency of MTurk for music similarity judgment. Each HIT contained 13–15 pairs of music samples. Workers listened to given pairs of samples and rated them on a 3-point scale (not similar, somewhat similar, and very similar). Each task contained one duplicated pair so that task results containing different answers for the same pairs were rejected. Moreover, tasks that were completed too quickly were also rejected.

Mandel et al. (2010) used MTurk for associating tags to songs. They used 925 10-s song clips. Workers had to provide 5–15 tags for each clip in five categories. Quality control was performed by rejecting tasks based on the length of words, size of vocabulary, or the frequency of stop words. The overall cost spent was approximately $100, so the average cost per accepted HIT can be estimated as at least $0.03.

Speck et al. (2011) used MTurk to annotate the emotional mood of songs. Each HIT contained 11 30-s song clips, including one dummy clip. Workers had to annotate the emotion that they felt on the two-dimensional arousal–valence (A–V) space while listening to each clip. Two clips were identical, so the tasks showing largely different results for that pair were rejected. Some other tasks in which workers did not seem to understand the instruction were also rejected.

These experiments were suitable to be conducted on MTurk, because each of them could be divided into small, distinct tasks and concealing their objectives was not important. In contrast, our experiment could not be constructed with separate, well-defined tasks, and natural music listening situations, which are difficult to be implemented within MTurk, were important to verify the music recommendation algorithm.

Because our experiment did not use MTurk, we need to define which data should be regarded as “accepted HITs” or “rejected HITs” for comparison with the aforementioned experiments. Our experiment interface gathered participants’ actions between transitions of song pairs (i.e., listening until the end and pressing the skip button). During the experiment, we implemented the following data validation mechanisms to reject untruthful responses (Choi and Lee, 2014).

• The system occasionally made the “wrong” recommendation. If the user kept listening without skipping, the actions of the user were filtered out.
• The system occasionally showed a dialog box containing a “continue” button. If the user did not press the button for a long time, the recent actions of the user were filtered out.

Only the actions that passed these validation mechanisms were used to improve the recommendation algorithm. Therefore, we consider these actions accepted HITs and the rest rejected HITs. The cost per accepted HIT in our experiment is also estimated based on the overall cost paid for the Facebook advertisement.

From the table, it can be concluded that our experiment has reasonable efficiency compared to the MTurk-based experiments. First, our experiment was able to recruit a larger number of participants than the other experiments. Moreover, the estimated cost per accepted HIT in our experiment is within the range of those of the other experiments even though our experiment rejected a larger number of tasks. Here, it should be noted that the HITs of each experiment have different complexities. The time complexity of the HIT in our experiment is similar to (or slightly less than) that in the experiment of Speck et al. (2011) where a HIT contained 11 30-s song clips. However, the cost per accepted HIT is much less in our case, which shows the efficiency of our crowdsourcing strategy. Although Mandel et al. (2010) did not mention the completion time per HIT, each HIT of the experiment contained only one 10-s song clip, which is very simple, and thus, the cost per accepted HIT is relatively low in comparison to that of our experiment.

### Advantages and Disadvantages of Social Networks for Crowdsourced Experiments

Based on our experiment and the analysis presented above, we find both advantages and disadvantages of using a social network to recruit participants for crowdsourcing-based experiments.

First, the experimenters can easily analyze the demographic characteristics of workers or trace them over time. Most social networks support Application Program Interfaces (APIs) to retrieve the basic information and interests of each user under authorized permission. This allows researchers to categorize collected data with respect to various aspects of personal information, which may be beyond country, age, or gender. In addition, those who participate in the experiment repeatedly can be easily traced.

In addition, social networks enable researchers to choose motivations other than financial reward, including enjoyment and implicit work. These types of motivation do not require direct payment to users. Participants are encouraged to join the experiment voluntarily, and those who wish to participate only for the money are excluded. Moreover, the experiment can proceed as though it is a real service, which allows observation of participants’ natural responses without bias.

Moreover, social networks allow experimenters to communicate with workers bilaterally, while most MTurk-based experiments implement only one-way feedback functions (Mandel et al., 2010; Speck et al., 2011). For example, our experiment used a Facebook page to communicate with and receive feedback from participants. Some users may have difficulty participating in experiments due to their particular environmental configurations, such as operating systems, network states, and web browsers. However, one-way feedback may not be sufficiently effective to identify and resolve such problems. Communication in both directions enables researchers to deal with unexpected errors or problems in the experiment in a timely manner, which is critical for ensuring the quality of data.

Finally, attracting participants via social networks is suitable for long-term crowdsourcing-based experiments. If the Facebook page or Twitter account for an experiment is maintained, the number of subscribers or followers can increase, and thus, more and more participants can be involved in the experiment as time goes on. Moreover, posting an article does not incur any charge, so the overall required cost to promote the experiment can be reduced in comparison to the case with one-time financial motivation when the experiment is operated for a long time.

However, there are also drawbacks of using social networks to attract participants. While the amount of money paid to participants can be easily defined and estimated in financial reward-based experiments, it may be rather difficult to estimate the cost required for advertisements to gather a sufficient amount of data in social networks. Moreover, privacy issues must be considered if detailed information of each user needs to be collected. Permission is required for retrieving some types of information, such as Facebook pages that people like and e-mail addresses, and people may not be willing to give such information readily (Vajda et al., 2011).

### Suggestions for Recruitment Based on Social Networks

In order to exploit the advantages of attracting participants via social networks, proper strategies are required, which are discussed below.

First, apart from paid advertisements, maintaining promotional pages such as the Facebook page is highly beneficial to increase the constant influx of participants. While advertisements are shown only for a limited period at a cost, articles on promotional pages can be permanently accessed at no cost. Moreover, most social network services offer so-called word-of-mouth features, including the “Like” button on Facebook and “Retweet” on Twitter. These features can facilitate the propagation of articles on the page for many people. Our experiment showed that the articles on the Facebook page were effective at attracting participants.

Next, advertisements placed in the same place as normal articles are more effective than those in other places. In our analysis, the newsfeed advertisement was shown to be better than the right-column advertisement on Facebook due to its higher CTR and lower CPC. In addition, the newsfeed advertisement offers socially connected features, such as the “Like” button of the Facebook page and a list of friends who like the page, which can increase interest in the advertisement.

Furthermore, adopting different strategies for different target user groups is important to enhance efficiency due to group-dependent behavioral patterns. According to our results, experiments performed directly on Facebook would engage women more easily than men, while Facebook-independent experiments that require users to go outside of Facebook would attract men better than women. In addition, a higher advertisement budget would be required to draw men than women and younger age groups than older age groups.

### Limitations of the Study

In these days, people use online social networks via mobile devices. However, the advertisement in our experiment targeted only the desktop environment because the validity of the experimental data gathered from the mobile environment was questionable; for instance, users may lose attention to the experiment easily in the mobile environment. Therefore, it is inconclusive whether our observations are valid in mobile-based experiments.

In addition, our advertisement target was limited to English-speaking countries. This was necessary because all the text in the experiment interface, advertisement, and Facebook page articles was English and the lyrics of the songs were also English. However, this might limit general applicability of our observations to non-English-speaking countries.

## Conclusion

In this paper, we presented and analyzed an approach of using a social network to attract participants for crowdsourced multimedia-involved behavioral testing. The analyzed results demonstrated that promoting the experiment on Facebook via a dedicated page and advertisements is effective to attract users. A comparison with other crowdsourcing projects having similar topics in multimedia showed that our approach has competitive efficiency and usability in recruiting a sufficient number of users and retrieving a high quality of data while keeping the cost for running the experiment comparably reasonable. In addition, we suggested methods of using online social networks as crowdsourcing media for achieving high efficiency. We anticipate that our analysis results will help researchers find efficient ways of gathering fine data from crowds.

## Conflict of Interest Statement

The authors declare that the research was conducted in the absence of any commercial or financial relationships that could be construed as a potential conflict of interest.

## Footnotes

1 Other social networks (e.g., Twitter) may be used, but we chose Facebook because it has the largest number of active users.
